# From HPV infection to cervical precancer: Molecular mechanisms, immune microenvironment and emerging technologies

**DOI:** 10.1007/s00018-026-06473-x

**Published:** 2026-10-16

**Authors:** Yubing Zhou, Yunxiao Ge, Kangdong Liu, Hui Liu, Zigang Dong

**Affiliations:** 1 Department of Pathophysiology, School of Basic Medical Sciences, College of Medicine, State Key Laboratory of Metabolic Dysregulation & Prevention and Treatment of Esophageal Cancer, Zhengzhou University, Zhengzhou, 450001 Henan China; 2 China-US (Henan) Hormel Cancer Institute, No.127, Dongming Road, Jinshui District, Zhengzhou, 450008 Henan China; 3 Department of Physiology and Pharmacology, Karolinska Institutet, Stockholm, Sweden

**Keywords:** Cervical precancer lesions, Cervical intraepithelial neoplasia, Transformation

## Abstract

Cervical cancer remains a leading cause of cancer-related mortality among women globally, with persistent high-risk human papillomavirus (HPV) infection as the primary etiological factor. Cervical precancerous lesions, including high-grade squamous intraepithelial lesions and adenocarcinoma in situ, represent the critical window for intervention prior to invasive cancer development. However, additional factors such as immunosuppression, smoking, and microbiome composition also play crucial roles in the development of cervical precancerous lesions. While HPV vaccination, screening, and treatment have reduced cancer incidence, these must be complemented by personalized approaches that target the molecular mechanisms driving carcinogenesis. Key dysregulated tumor suppressors (e.g., p16) and signaling pathways (PI3K/AKT/mTOR, Wnt/β-catenin) highlight the need for advanced therapeutic strategies. The immune microenvironment also plays a critical role in cancer progression, suggesting potential for immunotherapy. Promising biomarkers like p16, Ki67, and microRNAs require integration with technologies such as single-cell RNA sequencing and spatial transcriptomics for deeper insights into tumor heterogeneity and microenvironment dynamics. Additionally, while deep learning models hold promise in improving diagnostic accuracy for cervical intraepithelial neoplasia (CIN), challenges such as data variability and interpretability need addressing. In conclusion, incorporating genomics, transcriptomics, immunotherapy, and artificial intelligence will enable more personalized and effective interventions, ultimately reducing the global burden of cervical cancer.

**Graphical abstract:**

**Figure:**
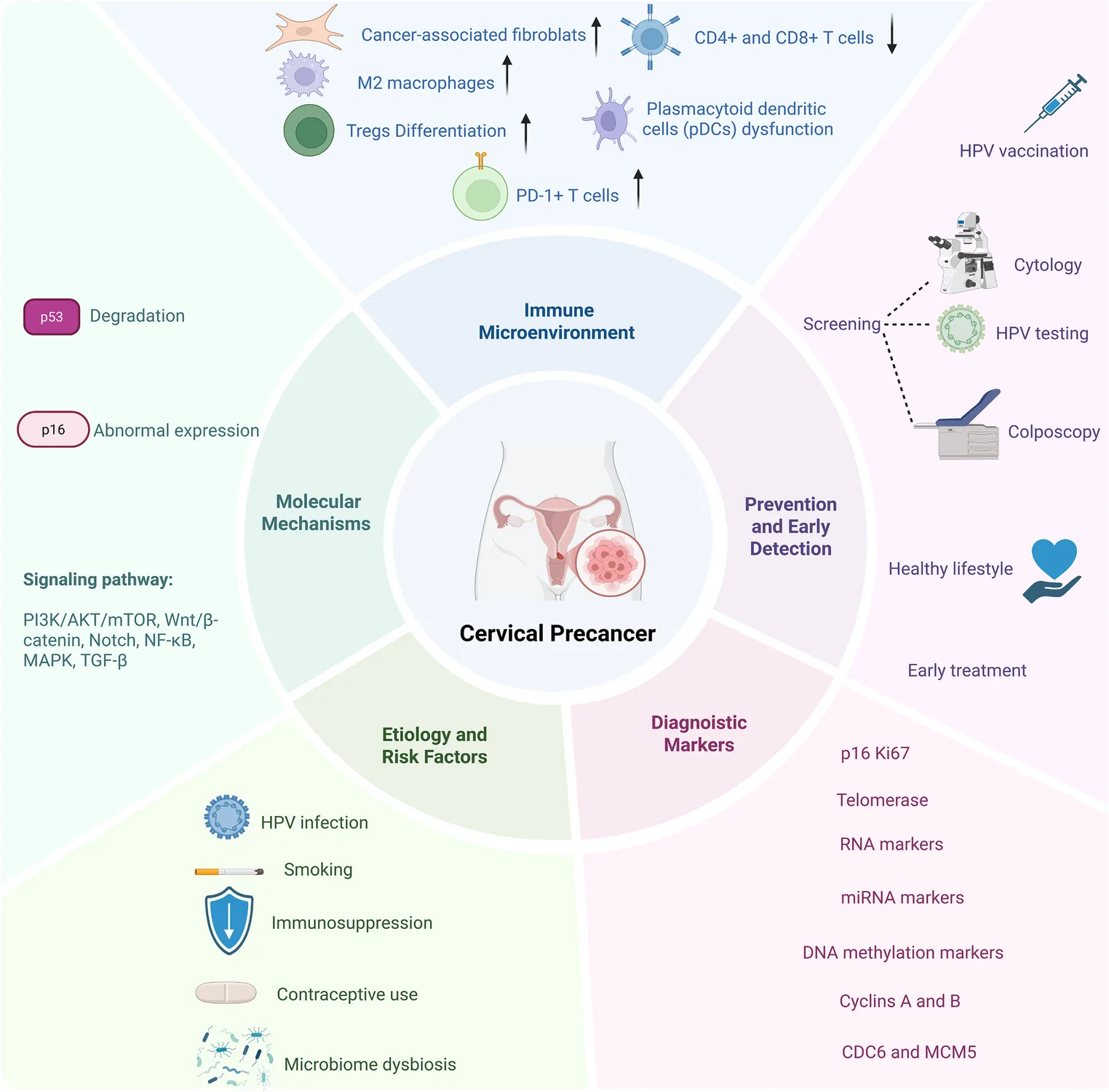

## Facts

Persistent high-risk HPV infection is necessary for cervical carcinogenesis, but cofactors (immunosuppression, smoking, and microbiome dysbiosis) modulate precancer initiation and progression.

Cervical precancer is the main prevention window, yet CIN2/HSIL is biologically heterogeneous with divergent regression and progression risks.

p16/Ki67 and core pathways (PI3K/AKT/mTOR and Wnt/β-catenin) link persistent infection to malignant transformation and define targets for precision prevention and therapy.

Immune microenvironment dysfunction enables HPV persistence and promotes progression, supporting immunomodulatory strategies in precancer.

microRNAs, single-cell/spatial transcriptomics, and deep learning are advancing risk stratification and diagnosis, but heterogeneity, standardization, and interpretability limit clinical translation.

## Open questions

How do HPV persistence and cofactors converge to determine regression, persistence, or progression of cervical lesions?

Which integrated criteria best resolve CIN2/HSIL heterogeneity and identify lesions requiring treatment versus surveillance?

Which actionable nodes in PI3K/AKT/mTOR and Wnt/β-catenin causally drive precancer progression, and can they be targeted safely?

Which immune-contexture features predict outcomes, and can immunotherapy be effective at the precancer stage?

How can single-cell/spatial multi-omics and deep learning be standardized into generalizable, interpretable clinical workflows?

## Introduction

Cervical cancer ranks as the fourth most common cancer among women globally [1]. In 2022, an estimated 662,301 new cases and 348,874 cervical cancer-related deaths were reported worldwide. Cervical cancer remains a leading cause of mortality among gynecological cancers [2]. Persistent infection with high-risk human papillomavirus (hrHPV) is the principal etiological factor for cervical cancer [3], with viral, host, behavioral, and environmental cofactors further influencing disease progression [4].

Cervical precancerous lesions are characterized by abnormal proliferation of cervical epithelial cells, which may progress to malignancy. These lesions are commonly identified through cytological and histological examinations and are categorized based on the degree of cellular abnormality [5]. CIN is classified into three grades encompassing CIN1 (mild dysplasia), CIN2 (moderate dysplasia), and CIN3 (severe dysplasia and carcinoma in situ). CIN1 represents a histopathological manifestation of HPV infection, which may undergo spontaneous regression [6]. CIN2 comprises a spectrum of lesions with varying propensities for progression to malignancy. CIN3 is the most clinically significant lesion and is widely regarded as the optimal surrogate endpoint for cervical cancer in screening and vaccination trials. CIN2 and CIN3 are generally classified as high-grade lesions; however, their biological behavior differs, with CIN2 showing greater heterogeneity in its potential for regression, persistence, or progression [7]. An alternative classification system, the squamous intraepithelial lesion (SIL) system, divides cervical lesions into low-grade squamous intraepithelial lesion (LSIL, typically corresponding to CIN1) and high-grade squamous intraepithelial lesion (HSIL, typically corresponding to CIN2 and 3).

Early screening and prevention are pivotal in reducing the global incidence and mortality rates of cervical cancer [8], 9]. HPV vaccination has been established as an effective strategy for mitigating cervical cancer development [10]. Extensive research has demonstrated that regular cervical cancer screenings, including cytology and HPV testing, enables early detection of cervical intraepithelial neoplasia, facilitating timely intervention before progression to malignancy [11]. Despite these advances, important clinical challenges remain. In particular, the heterogeneous natural history of CIN2, which may regress, persist, or progress, complicates individualized risk assessment and management [12]. Although targeted immunotherapeutic approaches, particularly therapeutic HPV vaccines, have been evaluated in clinical trials for CIN2/3, VGX-3100 achieved histopathological regression in 49.5% of treated women versus 30.6% receiving placebo in a phase 2b trial. However, their clinical efficacy remains modest and their implementation is limited; thus, they have not yet replaced conventional lesion-directed management [13, 14]. In this review, we first explore etiology, regulatory mechanisms and prevention of cervical precancerous lesions. We then systematically review the biomarkers associated with cervical precancerous lesions. Finally, we examine the applications of sequencing technologies and machine learning in cervical precancerous lesions and highlight future directions in this field.

## Etiology of cervical precancerous lesions

**Fig. 1:**
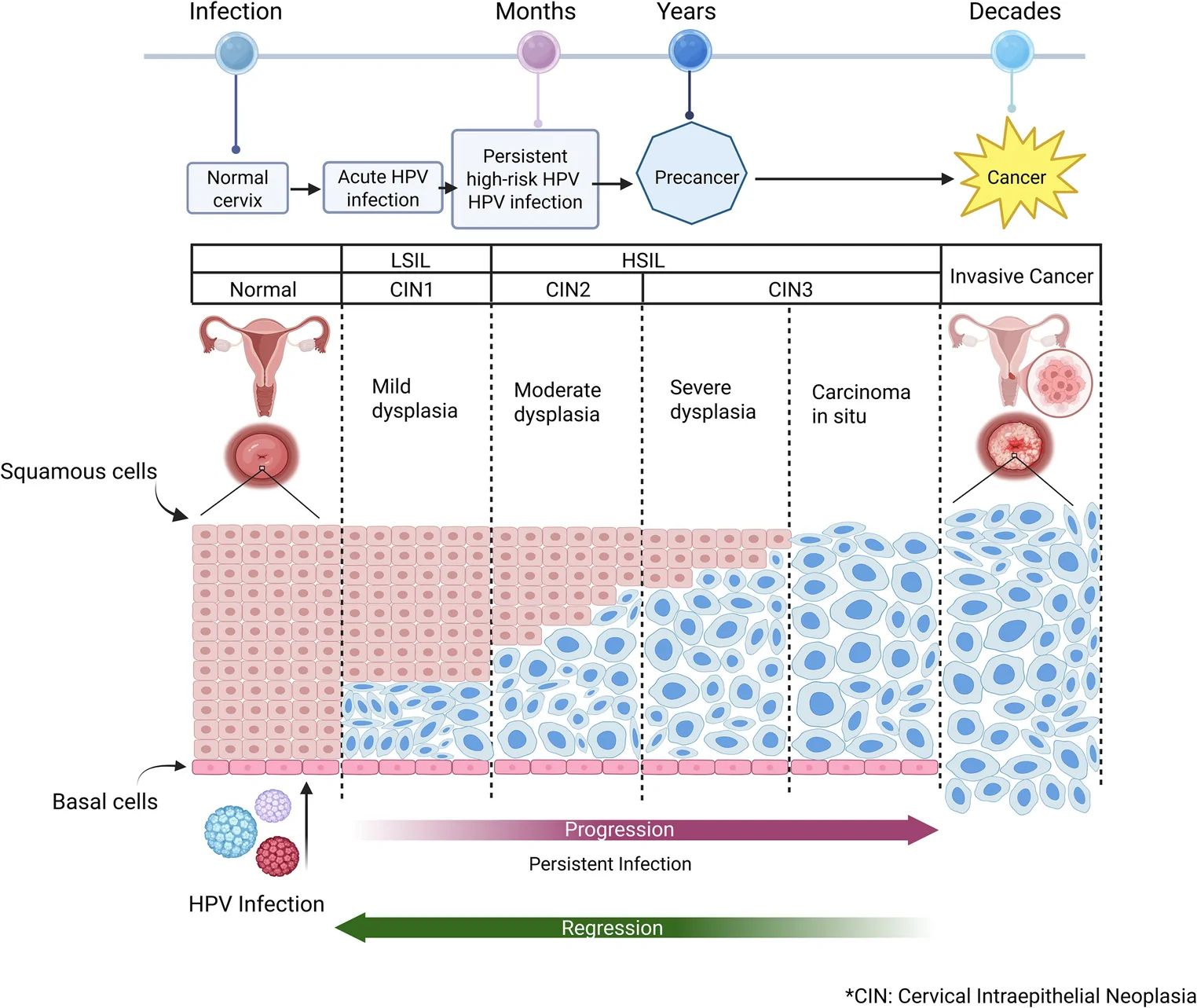
HPV infection and stage-wise progression of cervical cancer. The cervical intraepithelial neoplasia (CIN) scale is classified into CIN1 (mild dysplasia), CIN2 (moderate dysplasia), and CIN3 (severe dysplasia and carcinoma in situ). CIN is a pre-cancerous condition which is usually caused by persistent HPV infection of the cervix. Treatment may be needed to prevent development into cancer in the early stage

Human papillomavirus is implicated in approximately 5% of human cancers, notably cervical and oropharyngeal cancers [15, 16]. HPVs are recognized as the principal etiological agents of approximately 99% of cervical lesions [17]. HPV, a small DNA virus primarily transmitted through sexual contact, can infect various epithelial tissues, including skin and mucosal membranes [18, 19]. More than 120 HPV types have been identified, with approximately one-third exhibiting tropism for the genital squamous epithelia [17]. HPV can be classified into low-risk and high-risk types according to their oncogenic potential [18]. Twelve HPV types, including HPV16, 18, 31, 33, 35, 39, 45, 51, 52, 56, 58, and 59 are classified as high-risk HPV (hrHPV), designated by the IARC as Group 1 carcinogens [20]. HPV16 and HPV18, the two most prevalent hrHPV types, are associated with proliferative or neoplastic cervical intraepithelial neoplasia lesions that may progress to malignancy if left untreated [21] (Fig. 1). Approximately 70% of cervical cancer cases worldwide are collectively attributable to HPV types 16 and 18 [17, 22]. Low-risk HPV types, such as HPV6 and HPV11, are rarely linked to malignancy but can cause benign lesions, including genital warts [18].

HPV infection represents one of multiple risk factors for CIN. Most HPV infections are spontaneously resolved by the host immune system within months to years [23]. However, in 10–20% of women, HPV infection persists, which constitutes a prerequisite for progression to CIN [24, 25]. The likelihood of viral clearance depends on the duration of HPV infection. Factors influencing the persistence and progression of HPV include immunodeficiency, notably among HIV-positive women and transplant recipients, as well as viral type [26, 27]. However, other factors, including sexual and reproductive behaviors [28], recent use of oral contraceptives [29–31], smoking [32, 33], and Chlamydia trachomatis infection, have been implicated as contributing factors [34–36]. Cervical intraepithelial neoplasia progression is associated with increased vaginal microbiome diversity, which may modulate viral persistence and disease progression [37].

## Molecular mechanisms of cervical precancerous lesions

### Gene mutations and expression regulation

Mutations and dysregulation of key tumor suppressor genes facilitate the transition from normal to precancerous states. In cervical precancerous lesions, tumor protein p53 (p53) and cyclin-dependent kinase inhibitor p16 (p16) are critical tumor suppressor genes whose mutations and dysregulation are key drivers of disease progression. The p53 gene, widely recognized as the “guardian of the genome,” regulates cell cycle arrest, senescence, genome stability, DNA repair, and apoptosis [38, 39]. However, in cervical precancerous lesions, the p53 protein undergoes ubiquitin-mediated degradation mediated by the E6 oncoprotein of hrHPV types (HPV16 and HPV18) [40, 41]. Consequently, p53’s growth-arrest and apoptosis-inducing activities are compromised [42], enabling damaged cells to evade immune surveillance and sustain proliferation [43].

**Fig. 2:**
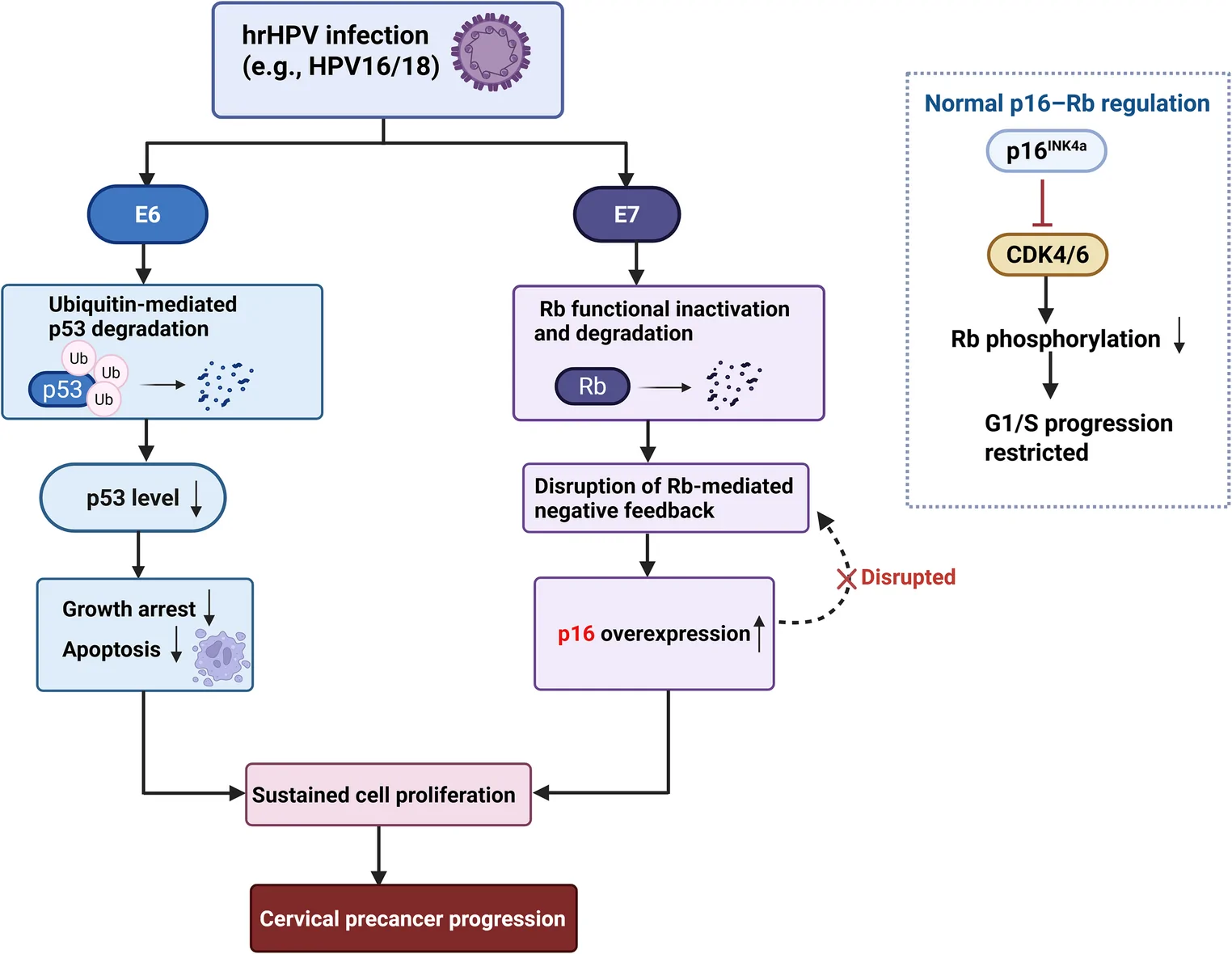
hrHPV E6/E7-mediated dysregulation of the p53 and Rb–p16 pathways in cervical precancerous lesions. E6 promotes ubiquitin-mediated p53 degradation, impairing growth arrest and apoptosis, whereas E7 induces Rb inactivation and degradation, disrupts Rb-mediated negative feedback, and results in p16^INK4a^ overexpression. Because the Rb pathway is functionally bypassed by E7, elevated p16^INK4a^ fails to restore effective cell-cycle control, ultimately promoting sustained cell proliferation and cervical precancer progression

p16 acts as a tumor suppressor in most cells. Typically, p16 binds directly to CDK4 and CDK6 and inhibits their kinase activity, thereby preventing Rb phosphorylation and restricting cell-cycle progression through the G1/S checkpoint [44]. However, in cervical precancerous lesions, HPV oncoprotein E7 promotes the functional inactivation and degradation of retinoblastoma protein (Rb), disrupting Rb-mediated negative feedback and resulting in p16 overexpression. Because the downstream Rb pathway is functionally bypassed by E7, elevated p16 does not restore effective cell-cycle control but instead serves primarily as a surrogate marker of HPV-mediated cell-cycle dysregulation [45] (Fig. 2). Numerous studies have demonstrated that p16 is overexpressed in a high proportion of preneoplastic lesions and nearly all cervical carcinomas, establishing it as a potential biomarker for identifying HR-HPV-infected low-grade lesions [46]. Accordingly, p16 immunohistochemistry is widely used in diagnostic pathology to aid the evaluation of CIN and other cervical lesions [47].

### Abnormalities in cell signaling pathways

**Fig. 3:**
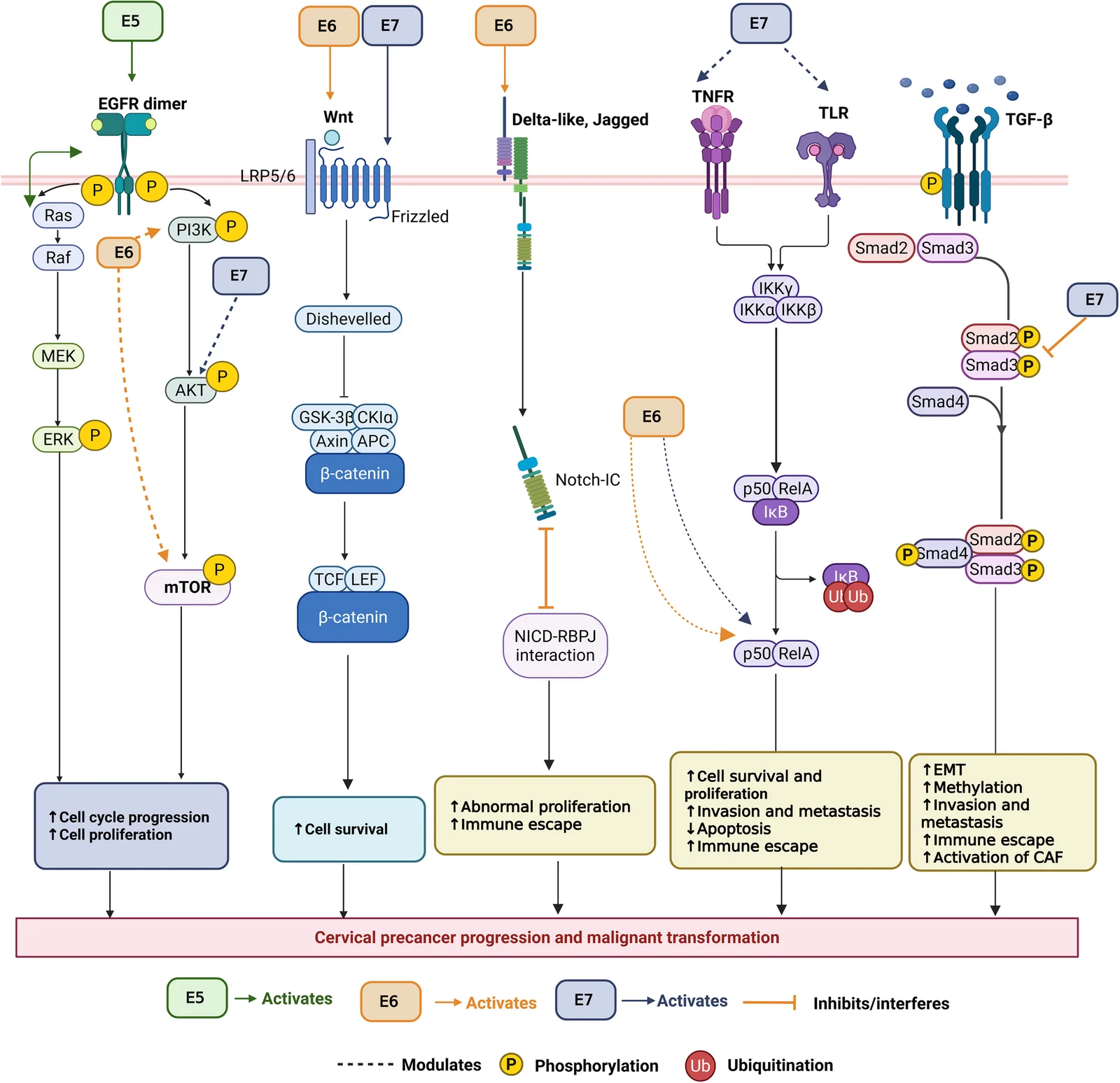
HPV oncoprotein-mediated dysregulation of key signaling pathways during cervical precancer progression. HPV E5, E6, and E7 modulate the PI3K/AKT/mTOR, MAPK, Wnt/β-catenin, Notch, NF-κB, TGF-β/Smad pathways, thereby promoting malignant phenotypes and cervical precancer progression toward malignant transformation. EGFR: epidermal growth factor receptor, RAS: rat sarcoma, RAF: rapidly accelerated fibrosarcoma, MEK: mitogen-activated protein kinase kinase, ERK1/2: extracellular signal-related kinase 1/2, PI3K: phosphoinositide 3-kinase, AKT: protein kinase B, mTOR: mammalian target of rapamycin complex, LRP5/6: low-density lipoprotein receptor-related protein 5/6, GSK3β: glycogen synthase kinase 3β, CKIα: casein kinase Iα, TCF/LEF: T-cell factor/lymphoid enhancer factor, APC: regulator of WNT signaling pathway, TNFR: tumor necrosis factor receptor, TLR: toll-like receptors, IKK: IκB kinase, IκB family (IκBα, IκBβ, and IκBγ), NF-κB1(p50), TGF-β: transforming growth factor β, SMAD family (Smad2, Smad3, Smad4), NICD: notch intracellular domain, RBPJ: recombination signal binding protein for immunoglobulin

In the progression of CIN, abnormalities are observed in several cellular signaling pathways (Table 1; Fig. 3). Dysregulated signaling pathways drive malignant transformation and promote tumor progression [48]. During malignant transformation, the major viral oncoproteins, E6 and E7, target the cellular tumor suppressors p53 and Rb, respectively, promoting proliferation and inhibiting apoptosis. Beyond their canonical effects on p53 and Rb, HPV oncoproteins E5, E6 and E7 modulate multiple oncogenic and immune-related signaling networks, as discussed below. Additionally, E6 and E7 modulate the immune response against infected cells through suppression of interferon (IFN) expression and signaling [49, 50].

**Table 1:** Abnormalities in Cell Signaling Pathways

Signaling pathway	Mechanism	Function	Therapeutic targeting and current evidence
PI3K/AKT/mTOR	HPV E6/E7 activate PI3K/AKT/mTOR signaling; E7 promotes AKT activation, E6 modulates PI3K/AKT and mTOR signaling, and HPV16 E5 activates EGFR and downstream PI3K/AKT signaling.	Promotes cell growth, survival, proliferation, metabolism, and carcinogenesis.	Rapamycin inhibits HPV-positive cervical cancer xenograft growth; PI3K/mTOR inhibitors suppress cervical cancer cell proliferation and tumor growth in preclinical models. PI3K inhibition can also enhance NaBT-induced cytotoxicity in HeLa cells.
Wnt/β-catenin	HPV16 E6 enhances E6AP-dependent β-catenin/TCF transcription, while E6/E7 may promote β-catenin accumulation and Wnt transcriptional activity. Crosstalk with Notch signaling may also occur.	Organogenesis, stem cell maintenance, cell survival, and tumor progression.	No targeted therapy has been approved for cervical precancerous lesions; Wnt inhibitors such as LGK974 remain under investigation.
Notch	HPV E6 interferes with NICD–RBPJ-dependent transcription and disrupts downstream Notch signaling; additional regulation occurs through miRNAs, epigenetic mechanisms, and pathway crosstalk.	Proliferation, differentiation, apoptosis, immune escape, and regulation of cell fate.	γ-secretase inhibitors such as MK-0752 have shown activity in early-phase studies of solid tumors, but their efficacy in cervical precancerous lesions remains unestablished.
NF-κB	HPV E6/E7 modulate NF-κB signaling, while persistent inflammatory signaling can promote pathway activation.	Promotes cell survival, proliferation, inflammatory responses, immune evasion, EMT, invasion, and metastasis.	NF-κB inhibitors such as bortezomib have shown preclinical activity in cervical cancer cells, but efficacy in cervical precancerous lesions remains unestablished.
MAPK	HPV16 E5 potentiates EGFR-mediated MEK/ERK1/2 signaling; persistent inflammatory stimulation can also dysregulate MAPK signaling.	Cell proliferation, cell-cycle progression, inflammatory signaling, impaired DNA damage repair, and increased mutation risk.	MEK inhibitors such as trametinib have activity in selected MAPK-driven cancers, but their efficacy in cervical precancerous lesions has not been established.
TGF-β	HPV16 E7 interacts with Smad2/3/4 and impairs Smad-mediated transcription, attenuating TGF-β growth-suppressive signaling; later TGF-β activity can promote EMT and immune evasion.	EMT, epigenetic and post-translational regulation, invasion and metastasis, immune evasion, and CAF activation.	TGF-β pathway inhibitors such as galunisertib have been evaluated in other malignancies, but clinical evidence in cervical precancerous lesions remains limited.

#### PI3K/AKT signaling pathway

HPVs engage with key upstream signaling pathways, including growth factor receptors, Notch receptors, Ras and PIK3CA genes, facilitating host cell survival and proliferation. This interaction drives carcinogenesis through activation and modulation of the PI3K/AKT/mTOR pathway [51]. The PI3K/AKT/mTOR pathway orchestrates multiple cellular events, particularly cell growth, survival, proliferation, and metabolism, influencing the initiation, progression, and clinical outcomes of various human cancers [52, 53]. HPVs exploit the host cell replication machinery to express E6/E7/E5 oncoproteins, which collectively contribute to cellular immortalization and carcinogenesis [54–56]. E6/E7 oncoproteins not only suppress tumor suppressors p53 and Rb and decrease apoptosis [57–59], but also activate PI3K/AKT/mTOR signaling, including E7-mediated AKT activation and E6-mediated modulation of PI3K/AKT and mTOR signaling [51, 60, 61]. In addition, HPV16 E5 activates EGFR, PI3K/AKT and MEK/ERK1/2 signaling [62]. Collectively, these mechanisms drive cell proliferation, thereby promoting carcinogenesis [63–66]. In preclinical models of HPV-positive cervical cancer, rapamycin significantly delayed xenograft growth, whereas the addition of the EGFR tyrosine kinase inhibitor erlotinib further enhanced tumor growth inhibition in some, but not all, models [67]. Consistent with these findings, our recent study further demonstrated that inhibitors targeting the PI3K/AKT/mTOR pathway reduced cervical cancer cell proliferation and colony formation in vitro, while PI3K and mTOR inhibition also suppressed tumor growth in cervical cancer cell-derived xenograft (CDX) models [68]. In addition, PI3K inhibition has been reported to enhance sodium butyrate (NaBT)-induced cytotoxicity in HeLa cervical cancer cells [69]. These findings provide preclinical evidence that the PI3K/AKT/mTOR pathway is pharmacologically targetable in HPV-associated cervical cancer. However, the therapeutic efficacy of targeting this pathway in cervical precancerous lesions remains to be established.

#### Wnt/β-catenin signaling pathway

The Wnt/β-catenin pathway comprises a family of proteins regulating essential cellular functions, including organogenesis, stem cell maintenance, cell survival, and mediates multiple intracellular signaling cascade [70]. Wnt activation is implicated in breast, lung, and hematopoietic cancers, contributing significantly to tumor recurrence [71–73]. Additionally, the Wnt pathway interacts with the Notch pathway, offering potential therapeutic targets for cancer treatment [74–76]. In the development of uterine cervical carcinoma, the Wnt/β-catenin pathway is frequently dysregulated [77]. HPV16 E6 contributes to Wnt/β-catenin dysregulation by enhancing E6AP-dependent β-catenin/TCF transcription, while E6/E7 may also promote β-catenin accumulation and Wnt transcriptional activity [78, 79].

Although several clinical trials have investigated agents targeting the Wnt and Notch pathways in hematologic and solid malignancies, no targeted therapies have yet been approved [80–83]. Currently, small molecule inhibitors targeting Wnt signaling, such as LGK974, have shown promise in preclinical studies, although their clinical application for cervical precancerous lesions is still under investigation [84].

#### Notch signaling pathway

The Notch pathway regulates key cellular processes, including proliferation, differentiation, and apoptosis. The abnormal proliferation of cervical cells is mediated by the Notch/p21/CDKs signaling pathway [85]. The Notch pathway modulates transcription factors such as Hes and Hey by activating RBP-JK, thereby influencing cell differentiation [86, 87]. Ma et al. demonstrated that Notch-1 modulates cervical cancer cell apoptosis through autophagy [88]. The Notch pathway mediates cell-cell communication through direct contact, influencing adjacent cell behavior. In HSIL, the HPV E6 protein disrupts downstream effector function in Notch signaling pathway. For example, it interferes with the interaction between notch intracellular domain (NICD) and recombination signal binding protein for immunoglobulin (RBPJ), disrupting the expression of Notch target genes. This disruption interferes with intercellular signal transduction, thus increasing the possibility of cellular transformation and carcinogenesis [89]. The activation of the Notch pathway could also influence the immune escape of tumors by modulating the immune cell function, including the polarization of T cells and macrophages [90, 91]. Additionally, the Notch pathway may also affect the HSIL malignant progression to ICC by miRNA regulation, epigenetic regulation, and cross regulation with other pathways [92, 93]. γ-secretase inhibitors, such as MK-0752, have shown efficacy in blocking Notch signaling and suppressing tumor growth in solid tumors in phase I trial [94–96]. However, clinical validation in cervical precancerous lesions is still needed, and the applicability of these inhibitors for cervical precancerous lesions treatment remains uncertain.

#### NF-κB signaling pathway

Dysregulated activation of the NF-κB (nuclear factor kappa B) pathway is a pivotal pathway in the progression of HSIL to invasive cervical cancer (ICC) [97]. NF-κB promotes cell survival and inhibits apoptosis through upregulation of anti-apoptotic genes and cell cycle regulators. This drives the uncontrolled proliferation of damaged cells and contributes to tumorigenesis [98]. NF-κB also modulates the expression of numerous inflammatory factors and chemokines. These factors recruit immune cells to the tumor microenvironment (TME) but also promote tumor progression and immune evasion by modulating immune cell activity [99]. Furthermore, NF-κB induces epithelial-to-mesenchymal transition (EMT), thereby increasing tumor cell invasiveness and metastatic potential [100]. Additionally, HPV E6 and E7 oncoproteins modulate NF-κB signaling, which may contribute to HPV-mediated cervical epithelial cell transformation [101]. Preclinical studies have investigated NF-κB inhibition in cervical cancer cells. Bortezomib enhanced radiosensitivity in SiHa cells but not in HeLa cells [102], while a separate screening study identified Bortezomib among several NF-κB inhibitors that suppressed cervical cancer cell growth [103]. However, the therapeutic efficacy of NF-κB inhibition in cervical precancerous lesions remains to be established.

#### MAPK signaling pathway

The MAPK family, including P38 MAPK, extracellular ERK1/2 and JNK, comprises a family of highly conserved serine/threonine kinases that regulate diverse cellular functions and play a significant role in the malignant progression of HSIL to ICC [104]. The MAPK pathway, particularly the ERK branch, drives cell proliferation through activation of cell cycle progression and inhibition of cell cycle regulators [105]. HPV16 E5 can directly enhance MAPK signaling by potentiating EGFR-mediated activation of the MEK/ERK1/2 cascade, thereby promoting mitogenic signaling and cell proliferation [106]. HPV infection induces an inflammatory response, and under persistent stimulation by inflammatory factors such as IL-1, IL-6, and TNF-α, the MAPK pathway is frequently dysregulated [107]. In addition, the MAPK pathway autonomously regulates cellular inflammatory responses and interacts with multiple signaling pathways, including TGF-β, PI3K/AKT, and NF-κB [108, 109]. Dysregulation of the MAPK pathway impairs DNA damage repair and elevates the risk of gene mutations [110, 111]. Trametinib, a MEK inhibitor, has demonstrated clinical activity in selected MAPK-driven malignancies [112, 113]. However, to our knowledge, clinical trials evaluating trametinib specifically in cervical precancerous lesions have not been reported, and its therapeutic efficacy in precancerous cervical lesions remains to be established.

#### TGF-β signaling pathway

TGF-β, a dimeric cytokine ubiquitously expressed in various cell types, regulates cell growth, migration, immune responses, epithelial-to-mesenchymal transition, and extracellular matrix (ECM) remodeling [114, 115]. Among its five subtypes, TGF-β1, TGF-β2, and TGF-β3 are the most abundant isoforms in mammals [116]. HPV oncoproteins can interfere with the growth-suppressive arm of TGF-β signaling during early transformation. HPV16 E7 interacts with Smad2, Smad3, and Smad4 and impairs Smad-mediated transcription, thereby attenuating TGF-β-mediated growth inhibition [117]. In the progression from HSIL to ICC, TGF-β plays a key role in EMT, epigenetic modifications, post-translational modifications and immune evasion [118]. TGF-β induces Smad signaling to upregulate EMT-related genes (e.g., Slug and Snail), which regulate epithelial and mesenchymal markers, thereby enhancing invasion and metastasis [119]. Additionally, CDR1 as facilitate m6A methylation of SLUG mRNA by IGF2BP1 and enhance its stability, thereby driving cervical cancer metastasis [120–122]. As an immunosuppressive cytokine, TGF-β facilitates immune evasion by suppressing immune cell function, enhancing regulatory T-cell (Treg) differentiation, and inducing cancer-associated fibroblasts (CAFs) activation, thereby promoting tumor progression [120, 123, 124]. Inhibitors targeting TGF-β, galunisertib, have shown good efficacy in hepatocellular carcinoma, advanced rectal cancer in phase 2 study [125, 126], but clinical trials evaluating these agents in cervical precancerous lesions are still limited.

Other signaling pathways, such as JAK/STAT, Hippo, and Hedgehog, also play roles in the progression from HSIL to cervical cancer [127]. Clinical evidence for pathway-targeted therapy in cervical precancer remains limited; in a randomized phase II trial, celecoxib did not significantly improve histologic regression of CIN3 compared with placebo [128].

Collectively, these molecular alterations not only promote epithelial transformation but also reshape the local immune microenvironment. Dysregulated signaling pathways, including NF-κB and TGF-β, can modulate inflammatory responses, immune-cell function, and stromal activation, thereby facilitating immune evasion and lesion progression. These molecular–immune interactions provide a mechanistic basis for the immune microenvironment changes discussed below.

## Changes in the immune microenvironment

### Immune cells and immune suppression during cervical carcinogenesis

**Fig. 4:**
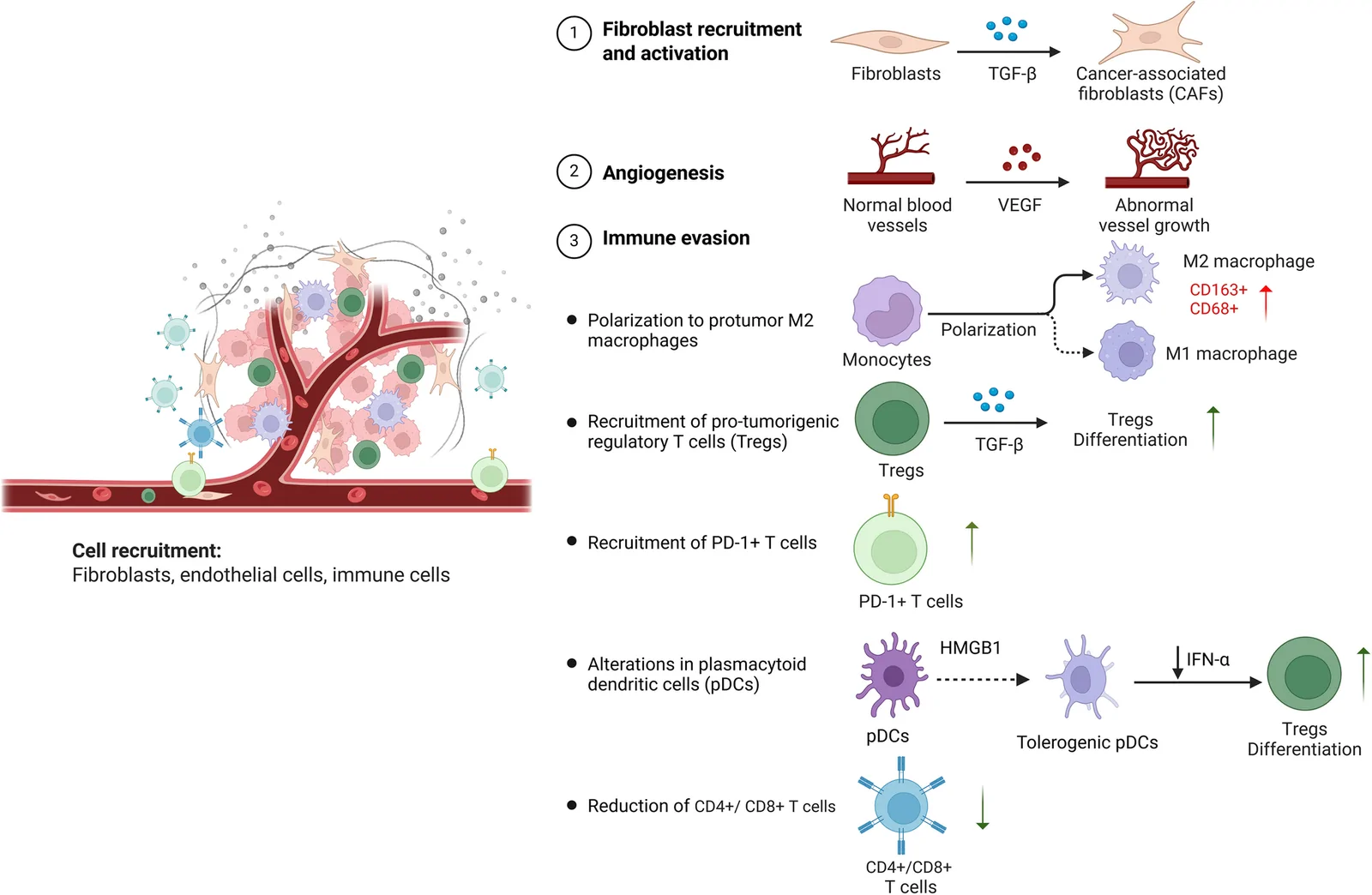
Immune microenvironment and immune evasion in cervical precancerous lesions. TGF-β-mediated activation of cancer-associated fibroblasts (CAFs) and VEGF-driven angiogenesis contribute to microenvironmental remodeling. Immune alterations include preferential recruitment of pro-tumor M2 macrophages, Treg differentiation, increased PD-1^+^ T cells, and reduced CD4^+^/CD8^+^ T-cell infiltration. In addition, HMGB1 released by neoplastic keratinocytes may induce a tolerogenic pDC phenotype with reduced IFN-α production, thereby promoting Treg differentiation and local immune evasion

In cervical neoplasia, the immune microenvironment is highly dynamic, evolving as lesions regress or progress to HSIL or cervical cancer (Fig. 4). The microenvironment comprises various cell types, including infected keratinocytes, immune cells, endothelial cells, pericytes, mesenchymal stem cells, and fibroblastic cells, which are interconnected through complex interactions [129].

TGF-β can activate CAFs, inducing the secretion of growth factors and cytokines such as VEGF. CAFs play a critical role in promoting tumor angiogenesis to supply oxygen and nutrients to the tumor, while fostering an immunosuppressed microenvironment. These mechanisms may facilitate the transformation from HSIL to cervical cancer [130].

Macrophages can be classified into two subtypes: type 1 macrophages (M1), which are pro-inflammatory and type 2 macrophages (M2), which are anti-inflammatory and associated with lesion progression [131, 132]. During disease progression, both CD68^+^ and CD163^+^ cell infiltrates significantly increase in the epithelium, particularly CD163^+^ cells, suggesting that M2 macrophages are preferentially recruited into the epithelium [133]. In the immune microenvironment, macrophage activation by HPV-infected tumor cells may lead to reduced antigen-presenting capacity and suppressed T cell proliferation, thereby promoting lesion progression [134, 135].

Plasmacytoid dendritic cells (pDCs) may also contribute to immune remodeling in cervical precancerous lesions. pDCs are recruited to cervical (pre)neoplastic lesions, where factors released by neoplastic keratinocytes, particularly high-mobility group box 1 (HMGB1), can impair pDCs maturation and reduce TLR9-induced IFN-α production, promoting a tolerogenic phenotype. Functionally altered pDCs may further facilitate Treg differentiation, thereby contributing to local immune tolerance and immune evasion [136].

The E6 and E7 antigens can be recognized by the adaptive immune system, leading to the elimination of HPV-infected cells [137, 138]. In persistent HPV infections, multiple immune escape mechanisms in the infected cell prevent an effective HPV-specific T-cell mediated immune response [138, 139]. TGF-β also prevents T cell infiltration into dysplastic tissue and T cell activation [140, 141]. Lower counts of infiltrating CD4^+^ and CD8^+^ T cells in HSIL reflect the compromise of the adaptive immune system by persistent hrHPV infection, thereby facilitating lesion persistence and progression to cervical cancer [142]. Additionally, PD-1/PD-L1 interaction observed in high-grade squamous intraepithelial lesion, characterized by an increased percentage of PD-1^+^ T cells compared to normal epithelium, may contribute to the suppressed immune microenvironment associated with disease progression [142, 143].

### The cervicovaginal microbiome as an immunomodulatory regulator

The cervicovaginal microbiome represents an important regulator of the local immune microenvironment during HPV infection and cervical carcinogenesis. Vaginal dysbiosis, characterized by decreased Lactobacillus abundance and increased microbial diversity, has been associated with persistent HPV infection and progression of CIN and cervical cancer [144, 145]. Longitudinal studies further indicate that cervicovaginal microbial composition is associated with the natural history of HPV infection, including viral clearance, persistence, and progression to CIN2+. In particular, Lactobacillus-dominant communities have been associated with HPV clearance or lesion regression, whereas increased microbial diversity and Lactobacillus depletion are linked to persistent infection and CIN progression [146, 147].

Vaginal dysbiosis may promote HPV persistence by inducing chronic inflammatory responses and impairing antiviral immune surveillance. Microbial imbalance can affect antigen presentation, T-cell activation, and immune cell recruitment, thereby reducing effective HPV clearance [144, 145, 148]. Microbial imbalance can activate inflammatory signaling pathways and create a chronic inflammatory niche that favors viral persistence and lesion progression [145, 149]. Furthermore, this inflammatory state may alter immune cell recruitment and function, thereby facilitating the persistence and progression of CIN and subsequent malignant transformation [144, 145, 148].

By promoting a local environment favorable to HPV persistence, microbiome dysbiosis may indirectly facilitate subsequent molecular alterations associated with malignant transformation. Among these alterations, HPV DNA integration represents an important event in cervical carcinogenesis, with integration sites frequently occurring at specific genomic hotspots [150]. Genome-wide profiling further suggests that HPV integration is non-random and may involve microhomology-mediated DNA repair mechanisms, potentially contributing to host genomic instability and oncogenic dysregulation [150].

Therefore, the cervicovaginal microbiome should be considered an essential component of the cervical immune microenvironment, linking microbial dysbiosis, chronic inflammation, immune dysfunction, HPV persistence, and the progression toward cervical carcinogenesis. Further studies integrating microbiome profiling with immune and genomic analyses are needed to clarify the mechanisms underlying cervical cancer development.

## Prevention of the transformation from cervical precancerous lesions to cervical cancer

**Fig. 5:**
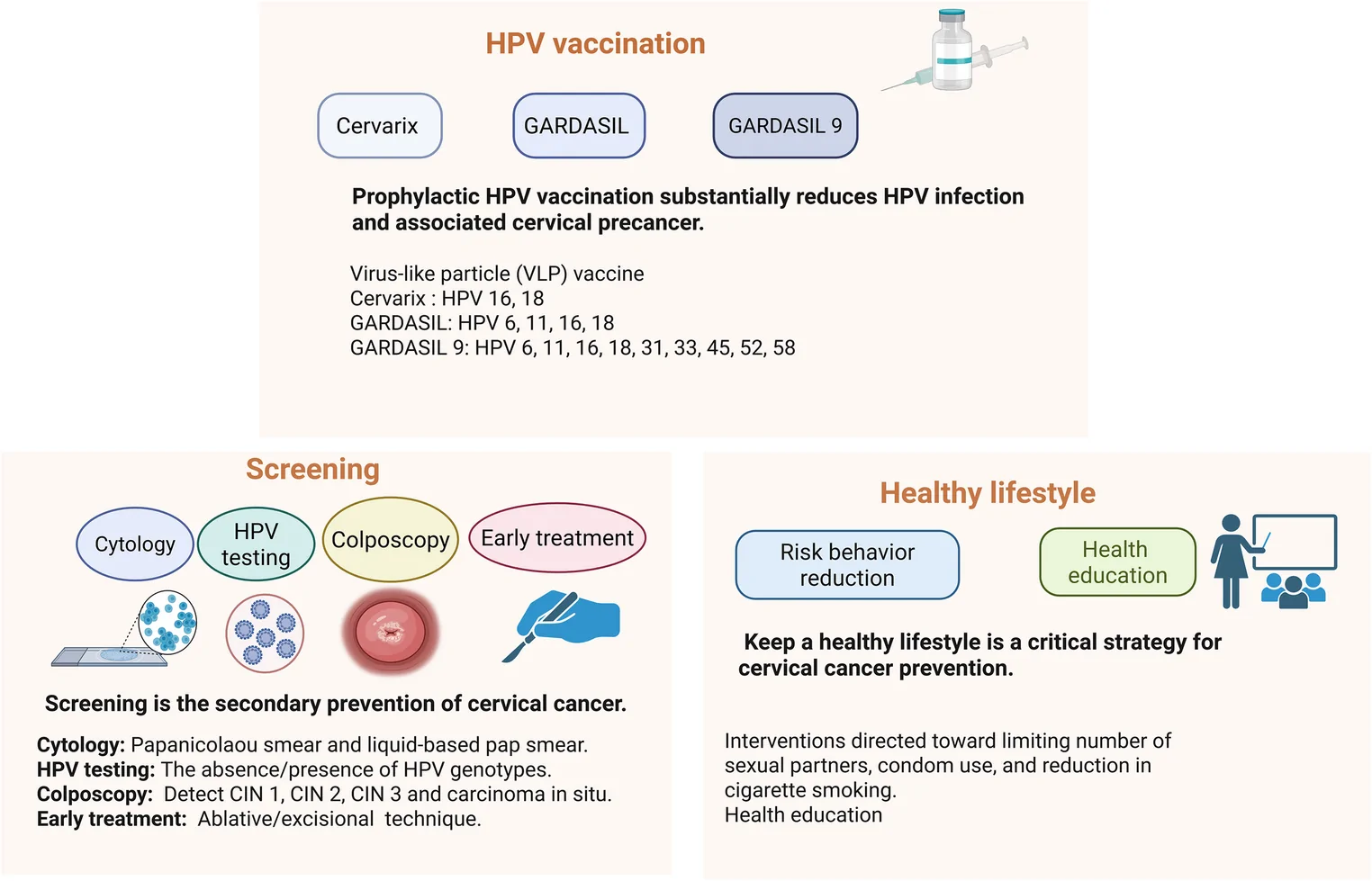
Prevention measures of cervical cancer. HPV vaccination, including cervarix, GARDASIL, and GARDASIL 9, is the effective primary prevention of cervical cancer. Screening is the secondary prevention of cervical cancer, which includes cytology, HPV testing, colposcopy, and early treatment of cervical precancerous lesions. Keeping a healthy lifestyle, including limitation of behaviors and health education is also effective for prevention of cervical cancer

Understanding the interplay among persistent HPV infection, molecular dysregulation, immune remodeling, and microbiome dysbiosis provides a mechanistic basis for developing preventive strategies across different stages of cervical carcinogenesis. The elimination of cervical cancer represents a significant public health challenge that necessitates comprehensive implementation strategies [151]. Cervical precursor lesions induced by chronic, persistent HPV infection, if left undetected and untreated, can progress to invasive cancer. Systematic cytology-based and HPV screening, combined with HPV vaccination programs and effective precancer treatment strategies, are critical to enhance the efficacy of prevention interventions [152] (Fig. 5).

### HPV Vaccination

Effective primary prevention of cervical cancer relies on HPV vaccination. Three HPV vaccines have been approved by the US Food and Drug Administration, namely GARDASIL 9 (9vHPV), GARDASIL (4vHPV), and Cervarix (2vHPV) [153]. These L1 virus-like particle-based vaccines induce type-specific neutralizing antibodies that prevent HPV infection. Since the introduction of the HPV vaccine in 2007, the prevalence of hrHPV infection has declined by up to 64% among adolescent women [154]. A meta-analysis reported approximately 95% efficacy of L1-based HPV vaccines against CIN1–3 [155].

By preventing infection with vaccine-covered oncogenic HPV types, prophylactic HPV vaccines reduce subsequent E6/E7-driven cellular transformation and the risk of cervical carcinogenesis [156, 157]. Furthermore, 9vHPV extends protection to five additional high-risk HPV types (31, 33, 45, 52, and 58), providing broader protection against HPV-related diseases [158]. These vaccines represent an effective tool in cancer prevention, especially in reducing the burden of cervical cancer and other HPV-related malignancies worldwide.

### Screening

Screening serves as the secondary prevention of cervical cancer, leading to well-documented declines in incidence and mortality [159]. The primary goal of cervical screening is to detect cervical intraepithelial neoplasms (CINs) as precancerous lesions and invasive cervical cancer [160]. Vaccines currently in use do not cover all HPV types associated with cervical cancer. Therefore, vaccinated individuals should adhere to recommended cervical cancer screening guidelines. Currently, three main screening approaches are utilized: cytology, HPV testing, and visual inspection [161]. Screening recommendations vary according to age and screening modality. For individuals aged 21–29 years, cervical cytology alone is recommended every 3 years. For those aged 30–65 years, recommended screening options include cervical cytology alone every 3 years, primary hrHPV testing every 5 years, or co-testing with hrHPV testing and cytology every 5 years. Individuals at higher risk of cervical precancer or cancer, including those with a history of abnormal cervical cells, precancer, cervical cancer, or immunosuppression, may require more frequent or individualized screening [162].

The Papanicolaou smear and liquid-based cytology are widely accepted screening tools [163]. HPV testing has been implemented as an adjunct to cytology or co-testing, following the FDA approval of the first hrHPV test designed specifically for primary cervical cancer screening [164]. Normal screening results include negative cytology and/or the absence of HPV. Abnormal results may include pathological cytology findings (e.g., atypical cells of undetermined significance, atypical squamous cells, suspicious for HSIL, atypical glandular cells, adenocarcinoma in situ) or the presence of specific HPV genotypes (e.g., hrHPV 16, hrHPV 18, other hrHPV types, LSIL, HSIL). To assess the risk of precancer, individuals with a positive cervicovaginal HPV result should undergo a Pap smear. If abnormal cells are detected on the Pap smear, patients typically proceed to colposcopy [161]. Colposcopy is a diagnostic procedure used to identify precancerous and cancerous lesions. It can also serve as an effective and safe alternative to cytology. Abnormal colposcopic biopsy results may include CIN1, CIN2, CIN3 and carcinoma in situ [165]. The final diagnosis of CIN is confirmed through histopathologic examination of biopsy specimens [166].

Epigenetic biomarkers, particularly DNA methylation, offer a promising approach for optimizing cervical pre-cancer screening. These biomarkers can detect early molecular changes in HPV-positive women with normal cytology, identifying those at higher risk of progression to cervical cancer. Methylation alterations in genes like the E6 and E7 oncoproteins of high-risk HPV strains play a key role in this process, making them valuable for risk stratification and triage after HPV screening. Integrating methylation testing with traditional HPV screening improves diagnostic accuracy, distinguishing between transient infections and those that are likely to lead to cancer [167, 168].

These biomarkers, especially when used in self-collected liquid biopsies, also enhance sensitivity and specificity in large-scale screening programs, particularly in resource-limited settings. They help prioritize women for closer monitoring and more frequent interventions, reducing unnecessary treatments for low-risk cases [169].

### Early treatment of cervical precancerous lesions

Cervical intraepithelial neoplasia can be managed using ablative techniques (e.g., cryotherapy or thermal ablation) or excisional techniques (e.g., large loop excision of the transformation zone or cold knife conization) [170–172]. The choice of treatment depends on the grade and extent of the disease [173]. The cure rate following excisional treatment of high-grade CIN is slightly higher than that achieved with ablative treatment [170]. Early treatment is an effective strategy to prevent the progression of CIN 2–3 to cervical cancer [170, 174].

### Healthy lifestyle

Maintaining a healthy lifestyle, including interventions such as limiting the number of sexual partners, consistent condom use, and reducing cigarette smoking, is a critical strategy for cervical cancer prevention [159, 175]. Health education interventions have the potential to reduce HPV transmission, thereby decreasing the incidence of cervical carcinoma [176].

## Markers of cervical dysplasia

**Fig. 6:**
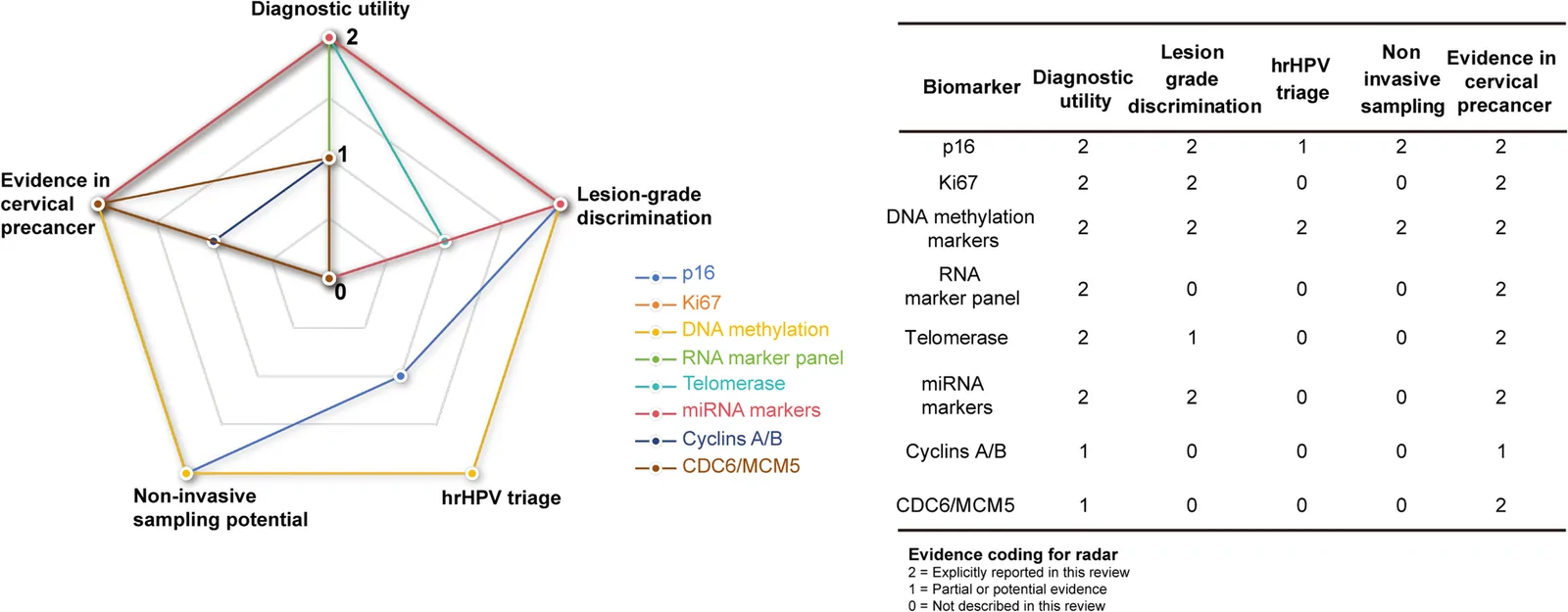
Comparative evidence profiles of diagnostic biomarkers for cervical precancerous lesions. Eight biomarker categories are compared across five evidence dimensions: diagnostic utility, lesion-grade discrimination, hrHPV triage, non-invasive sampling potential, and evidence in cervical precancer. Evidence was qualitatively coded as 2 (explicitly reported), 1 (partial or potential evidence), or 0 (not described) based on the studies summarized in this review. The radar chart reflects differences in the types and extent of reported evidence rather than direct head-to-head diagnostic performance; a score of 0 does not imply absence of diagnostic value

The identification of markers for cervical dysplasia has the potential to enhance clinical specificity in detecting cervical disease and reduce the need for unnecessary biopsies or surgical interventions [177]. These are several potential markers of cervical precancerous lesions (Table 2; Fig. 6). Numerous studies have demonstrated that the immunocytochemical detection of p16INK4a in cervical cytologic specimens serves as a reliable marker for cervical dysplasia and carcinoma [178, 179]. Generally, the proportion of p16-positive cases correlates strongly with the severity of cytologic diagnoses [180]. Researchers have also suggested that p16INK4a expression may aid in identifying low-grade cervical lesions associated with hrHPV types [181, 182].

**Table 2:** Cervical precancerous markers

Marker	Current application	Detection	Significance
Ki67	> CIN2 diagnosis, LSIL distinguishment from normal tissue	Immunohistochemical expression	Immunohistochemical marker for LSIL diagnosis
P16INK4a	Low-grade cervical lesions	Immunohistochemical expression and immunocytochemical detection	A reliable marker for cervical dysplasia and carcinoma
DNA methylation markers (CA10, DPP10, and HAS1 etc.)	CIN2/3 detection and cervical cancer risk prediction.	cervical scrapings, and urine	CIN2/3-specific methylation markers and hrHPV-positive women screening
RNA markers (claudin 1, minichromosome maintenance deficient 5 and 7, cell division cycle 6 homologue etc.)	CIN diagnosis	Cervical exfoliated cells	In alternative detection formats or in combination with other screening indicators
Telomerase	HGCIN and CSCC diagnosis	Cervical smear samples	A diagnostic biomarker for cervical dysplasia and carcinoma, diagnostic adjunct for cervical cytopathology
miRNA markers (hsa-miR-26b-5p, hsa-miR-191-5p, and hsa-miR-143-3p etc.)	CIN3 distinguishment from normal tissue	RNA in frozen tissue sections	Diagnostic markers for cervical premalignant lesions and cancer
Cyclin A and B	Expression of Cyclin A and B in Invasive adenocarcinomas > cervical glandular intraepithelial neoplasia	Immunohistochemical expression	Cervical glandular neoplasia diagnosis
CDC6 and MCM5	mRNA level of CDC and MCM5 in CIN, CSCC > normal cervical biopsies	RNA in FPPE tissues	Histopathological diagnosis of cervical dysplastic and malignant cells

Ki67 immunohistochemical expression demonstrates high sensitivity and specificity in diagnosing > CIN2 and distinguishing LSIL from normal cervical tissue [183, 184]. Therefore, Ki67 may serve as an immunohistochemical marker for diagnosing LSIL. A statistically significant correlation was observed between p16INK4a and Ki67 expression and histological grade (CIN1, CIN2, and CIN3) [185]. However, p16 was diffusely and strongly positive in some LSILs, and its practical application remains challenging [184].

The Illumina Infinium Methylation EPIC array revealed that methylation levels of individual markers (CA10, DPP10, and HAS1) gradually increased with lesion severity and were significantly elevated in CIN3 and carcinoma samples compared to normal samples [186]. These markers may discriminate among preinvasive CIN grades and predict cervical cancer risk. Genome-wide DNA methylation analysis identified new CIN2/3-specific methylation markers (ZSCAN1, ST6GALNAC5, ANKRD18CP, PAX2, CDH6, GFRA1, GATA4, KCNIP4, and LHX8) for detecting cervical neoplasia in cervical scrapings [187]. These newly identified markers could serve as a triage test for hrHPV-positive women in population-based screening [188]. Additionally, hrHPV DNA and DNA methylation testing in urine represents a promising approach for detecting cervical cancer and CIN2/3 lesions [189, 190].

RNA markers, such as claudin 1, minichromosome maintenance deficient 5 and 7, cell division cycle 6 homologue, antigen identified by monoclonal antibody Ki-67, and SHC SH2-domain binding protein 1, have been shown to form a panel that exhibits high sensitivity and specificity in diagnosing CIN [191].

Telomerase is widely expressed in a high proportion of high-grade squamous intraepithelial lesions and thus serves as a diagnostic biomarker for cervical dysplasia and carcinoma [190, 192]. The detection of telomerase activity in the cervix may provide insights into cervical carcinogenesis [193].

The miRNA markers hsa-miR-26b-5p, hsa-miR-191-5p, and hsa-miR-143-3p are associated with HPV status in patients and can distinguish CIN3 from normal cervical tissue [194]. Additionally, six oncogenic miRNAs (miR-20a, miR-92a, miR-141, miR-183*, miR-210, and miR-944) hold potential as diagnostic markers for cervical pre-malignant lesions and cancer [195].

Cyclin A and B expression are upregulated in neoplastic cervical lesions, and cyclin B is particularly valuable for diagnosing cervical glandular neoplasia [196].

CDC6 and MCM5 play critical roles in eukaryotic DNA replication. Several studies have demonstrated the potential of these proteins as molecular markers in the histopathological diagnosis of dysplastic and malignant cells [197].

Recent computational analyses have also identified lineage-associated molecular alterations, such as CRISP3 downregulation in cervical cancer, although their relevance to cervical precancerous lesions requires further validation [198].

Although these conventional molecular biomarkers have demonstrated diagnostic potential, they provide limited information on cellular heterogeneity and cell-specific interactions within precancerous lesions. Emerging single-cell and spatial technologies offer an opportunity to resolve these features at higher molecular and cellular resolution.

## Application of scRNA sequencing technology in the cervical precancerous lesions

**Fig. 7:**
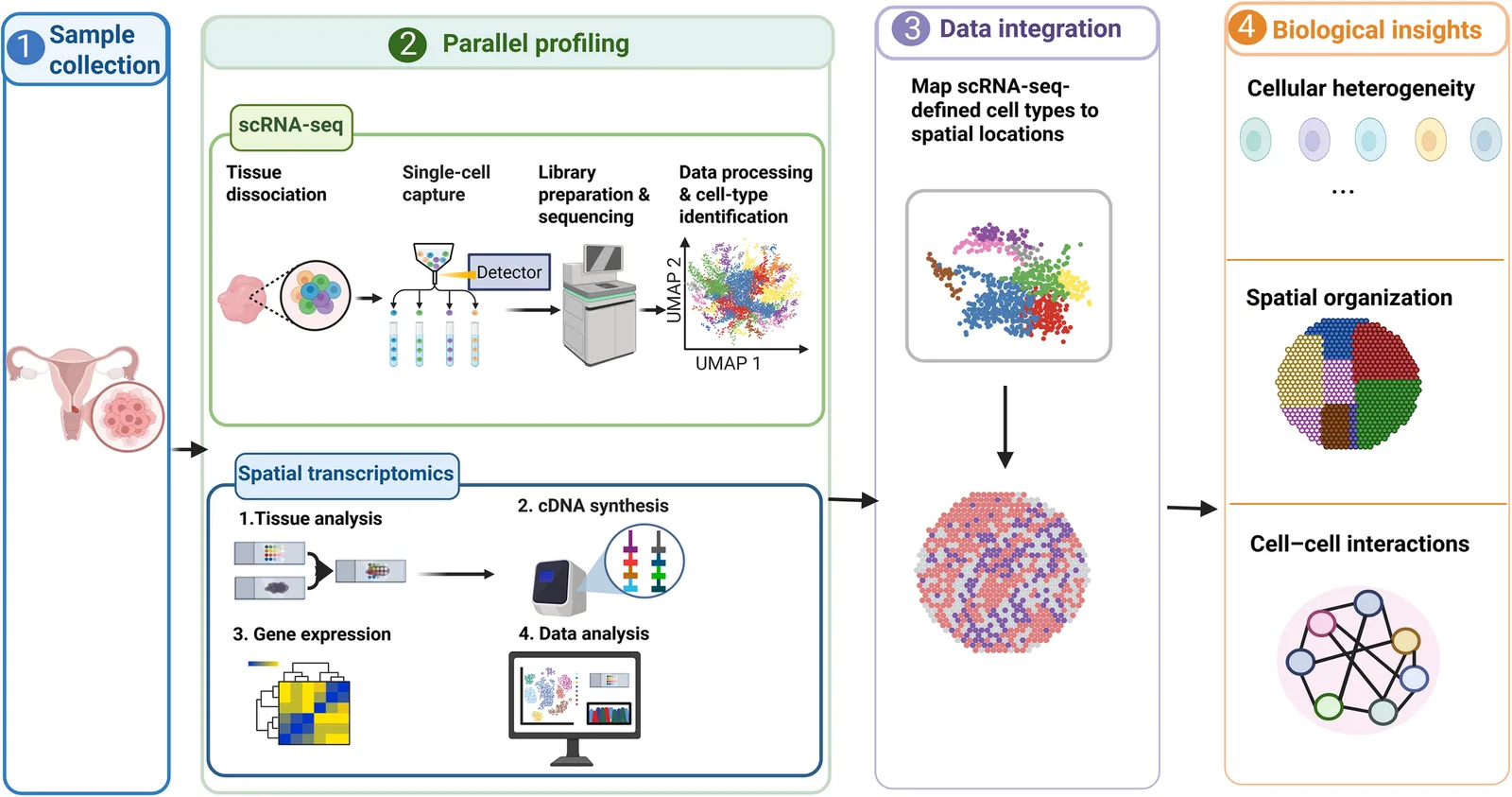
Integrated single-cell and spatial transcriptomics workflow. Parallel scRNA-seq and spatial transcriptomic profiling enables cell-type identification and spatial gene-expression mapping, respectively. Integration of these complementary datasets’ maps cell identities to their spatial locations, facilitating the characterization of cellular heterogeneity, spatial organization, and cell–cell interactions

Single-cell RNA sequencing (scRNA-seq) is a powerful tool for uncovering cellular heterogeneity and molecular characteristics in the TME in cancer research [199–201]. Recent progress in data analysis has shown that scRNA-seq can identify complex cellular subpopulations and predict intercellular crosstalk, enabling the identification of key ligand-receptor pairs involved in tumor progression [200, 202] (Fig. 7; Table 3). This technology provides an unparalleled molecular resolution, offering insights into various cancers, including breast and ovarian cancer [203, 204]. Importantly, single-cell RNA sequencing provides unique potential to advance the diagnosis, surveillance, and therapy of cancer [205].

**Table 3:** Comparison of scRNA-seq, spatial transcriptomics, and deep learning in cervical precancerous lesion research and clinical translation

Technology	Key strengths	Clinical applications and value	Major clinical limitations
scRNA-seq	Characterizes cellular heterogeneity and rare cell populations; identifies cell-specific transcriptional states and intercellular communication	Characterization of epithelial evolution and immune-cell states; candidate biomarker discovery, molecular risk stratification, and identification of potential therapeutic targets	High cost; stringent requirements for sample freshness and viability; specialized sample-processing and sequencing infrastructure; substantial computational and bioinformatics requirements; loss of spatial information
Spatial transcriptomics	Preserves spatial context of gene expression and tissue architecture; characterizes spatial cellular organization and interactions	Mapping of immune niches and spatial ligand–receptor interactions; identification of candidate spatial biomarkers and potential features for risk stratification	High cost; specialized tissue-processing and sequencing/imaging infrastructure; substantial computational requirements; platform-dependent resolution; limited prospective clinical validation
Deep learning	Automated and scalable analysis of cytological and colposcopic images; rapid feature extraction; potential for multimodal data integration	Computer-assisted screening and diagnosis, automated cytology/colposcopy classification, and integration with HPV and cytology testing	Dataset and device variability; limited generalizability; dependence on high-quality annotated datasets; limited interpretability; need for multicenter external validation

To investigate the structural heterogeneity and alterations in fibroblasts and endothelial cells in cervical cancer, scRNA-seq has been applied [206–209]. For instance, to elucidate CESC initiation and progression, Liu et al. conducted scRNA-seq analysis on samples of normal cervix, cervical intraepithelial neoplasia, early CSCC, and advanced CSCC [210]. Regarding the transcriptional evolution of epithelial cells during tumor initiation and progression, they observed that the expression of the malignant squamous cell marker (KRT17) and canonical proliferation markers (MKI67, CDKN2A, and TOP2A) increased from CIN to early and advanced CESC [211–214]. These molecular changes may provide candidate markers for early patient stratification; however, their diagnostic performance relative to conventional cytology-based screening requires further validation. Furthermore, intercellular communication analysis predicted that matrix-fibroblasts promote CESC proliferation, migration, and chemotherapy resistance primarily through the NRG1/ERBB3 pathway-an axis implicated in drug resistance in mutant BRAF melanoma [215] and prostate cancer [216], highlighting possible therapeutic intervention targets.

To reveal the altered characteristics of the TME in cervical carcinogenesis, Sheng et al. generated scRNA-seq profiles using cervical exfoliated cells, including normal cervix, low-grade squamous intraepithelial lesions, high-grade squamous intraepithelial lesions and cervical cancer [217]. They observed that, consistent with previous findings, exfoliated epithelial cells undergo progressive changes during cervical carcinogenesis [210]. Their analysis also identified serglycin (SRGN) and histone cluster 1 h1 family member C (HIST1H1C) as novel biomarkers associated with cervical cancer development, paralleling their roles in hepatocarcinogenesis, esophageal carcinoma, and nasopharyngeal carcinoma [218–220]. Importantly, LSIL samples, which contained abundant cytotoxic T cells and natural killer T cells, exhibited an activated immune phenotype, suggesting that immune-based early diagnostic approaches may be feasible. Additionally, lipid-associated macrophages may play an oncogenic role and represent candidate biomarkers associated with the progression from LSIL to HSIL, with potential implications for clinical follow-up strategies. The identification of SPP1-CD44 interactions associated with HSIL and CC underscores the potential of scRNA-seq-derived ligand-receptor networks for biomarker discovery and risk assessment. This study identified biomarkers for early detection and investigated the multicellular ecosystem in cervical carcinogenesis.

To reveal alterations in immune characteristics, Guo et al. expanded on these findings by constructing a comprehensive single-cell atlas using samples from HPV-negative normal, HPV-positive normal, HPV-positive HSIL, and HPV-positive cancer samples [221]. They found increased immune-suppressive potential and T cell dysfunction, which may serve as functional biomarkers to differentiate cancerous lesions from precancerous lesions. Specifically, plasmacytoid dendritic cells and macrophage polarization signatures were identified as potential markers for molecular diagnostic assays. When compared to conventional pathology, which primarily detects morphological changes, scRNA-seq offers higher-dimensional information on immune dysfunction and intercellular communication, potentially facilitating the differentiation between precancerous and malignant states.

Together, these findings highlight the potential of scRNA-seq as a promising platform for novel biomarker discovery, patient stratification, and early detection in cervical precancerous lesions. However, its clinical translation remains constrained by high costs, specialized sample-processing and sequencing infrastructure, and substantial computational and bioinformatics requirements [222].

## Application of spatial transcriptomics in cervical precancerous lesions

Spatial transcriptomics (ST) enables detailed characterization of the tissue microenvironment while showing potential translational value for biomarker discovery, risk stratification, and therapeutic target identification in cervical precancerous lesions.

ST complements scRNA-seq by preserving spatial information that is lost during tissue dissociation, thereby enabling transcriptomic profiles to be mapped within their native tissue architecture [221] (Fig. 7; Table 3). In melanoma, spatial transcriptomics has been utilized to create new prospects in cancer research [223, 224]. In cervical precancerous lesions, the spatial dimensions of entire transcriptomes and the TME have been systematically investigated. For instance, Guo et al. also revealed the spatial architecture of various cell subsets during the progression from HPV-infected normal cervix tissues to precursor lesions and cervical cancer [221]. They found CD8^+^ MAIT cells were enriched in HSIL tissues, including glands, HSIL foci, and connective tissues, playing a critical role in immune surveillance. Other studies have further characterized the spatial coexpression of ligand-receptor pairs across different stages of cervical carcinogenesis, from HPV infection and precancerous lesions to cervical cancer. By integrating scRNA-seq with ST, these studies spatiotemporally elucidated the mechanisms of HPV-related cervical carcinogenesis, including the malignant transition of epithelial cells and the remodeling of the immune microenvironment [225–227]. Collectively, these findings have advanced our understanding of HPV-related cervical carcinogenesis.

Spatial transcriptomics has provided new insights into the clinical landscape of precancerous cervical lesions by integrating molecular profiles with spatial context. Recent studies have shown that spatial mapping of the tumor microenvironment reveals immunosuppressive niches emerging from HPV infection to HSIL, highlights key immune subsets such as CD8⁺ MAIT cells, and identifies ligand-receptor interactions like SPP1-CD44 axis, MDK-SDC1 pathway that shape local immune regulation [221, 228, 229]. These findings highlight the potential of spatial transcriptomics to facilitate the identification of candidate biomarkers and molecular features that may contribute to risk stratification and therapeutic intervention in cervical precancerous disease.

Nevertheless, the clinical implementation of ST is currently constrained by its high cost, specialized tissue-processing and sequencing or imaging infrastructure, and substantial computational requirements [226]. Although scRNA-seq- and ST-derived candidate biomarkers have shown promise in exploratory studies, prospective clinical validation specifically in cervical precancerous lesions remains lacking [221, 226]. Larger prospective studies with adequate sample sizes and longitudinal follow-up are therefore needed to determine whether these biomarkers can reliably predict lesion persistence, regression, or progression and improve clinical risk stratification.

## Application of deep learning in the cervical precancerous lesions

It is increasingly evident that the visual identification of precancerous lesions by experienced nurses and doctors using a colposcope is often unreliable and inaccurate [230, 231]. Novel computer-based pattern recognition methods, commonly known as deep learning or machine learning, are becoming increasingly useful in diagnostic settings and playing a critical role in computer-assisted diagnosis [232]. Deep learning (DL) models have demonstrated promising diagnostic performance in cervical precancerous lesion detection. For example, automated visual evaluation achieved an AUC of 0.91 (95% confidence interval [CI] = 0.89 to 0.93) for detecting cervical precancer/cancer [233]. A DL-based colposcopy classification model achieved a sensitivity of 85.38%, specificity of 82.62%, and AUC of 0.93 for distinguishing positive (LSIL+) from negative cases [234]. DL models can automatically identify abnormalities in digital cytological images [235] and assist in colposcopy diagnosis and prognostic prediction of CIN [236–239]. Additionally, automated visual evaluation (AVE), a novel deep learning-based computer application, can predict cervical precancerous lesions [240]. Integrating colposcopy-based DL with cytology and HPV testing further improved screening performance, with the cross-modal integrated model achieving an AUC of 0.921 compared with 0.845 for the multiple-image DL model alone [241].

Deep learning has shown promise in diagnosing cervical precancerous lesions, but challenges remain in its clinical application. Data variability is a major issue, as differences in datasets from diverse populations or equipment can affect model performance and generalization. To address this, standardizing data collection and using techniques like transfer learning and data augmentation can help improve model robustness [242]. Another key challenge is model interpretability. Many DL models act as “black boxes”, making it difficult for clinicians to trust their predictions. Explainable AI (XAI) methods, such as attention mechanisms and feature visualization, are being developed to improve transparency and clinician confidence [243]. In a large population-based screening study involving more than 700,000 Chinese women, AI-assisted cytology showed higher sensitivity for detecting CIN2 + than manual cytology, supporting its potential application in large-scale cervical screening [244]. Notably, Bai et al. developed an attention-based AI-assisted cervical cytology screening system using a compact microscope for resource-limited settings, with attention maps highlighting lesion-related regions and improving model interpretability [245]. Overcoming these challenges by improving data standardization and integrating explainable AI is crucial for the effective clinical adoption of DL in cervical precancerous lesion detection [246].

## Conclusions and discussion

Cervical precancerous lesions represent a critical window of opportunity for the prevention of cervical cancer, which remains a significant global health challenge [247]. Persistent hrHPV infection is the principal driver of these lesions; however, interactions with host and environmental cofactors, including immunosuppression, smoking, and microbiome dysbiosis, may influence lesion persistence and progression. Future research should therefore move beyond identifying individual risk factors toward elucidating how viral, molecular, immune, and environmental alterations interact during cervical carcinogenesis. Based on the current evidence and remaining translational gaps, future research priorities can be broadly organized into four interconnected directions: molecular targeting, immune-based therapy, multi-omics biomarker development, and interpretable AI-assisted diagnosis.

### Molecular targeting

Aberrant molecular signaling represents a potential therapeutic vulnerability in cervical precancerous lesions. Although HPV vaccination and screening have substantially advanced cervical cancer prevention, additional therapeutic strategies are needed for established high-grade lesions. Dysregulation of tumor suppressors such as p53 and p16 and alterations in major signaling pathways, including PI3K/AKT/mTOR, Wnt/β-catenin, Notch, NF-κB, MAPK, and TGF-β, provide potential molecular targets for intervention. However, most of these molecular alterations have not yet been translated into clinically established targeted therapies for cervical precancerous lesions. Clinical trial evidence also remains limited, with available pathway-targeted interventions failing to demonstrate clear overall efficacy in cervical precancerous lesions. Future studies should distinguish alterations that merely correlate with lesion grade from those that causally drive lesion persistence or progression and evaluate actionable targets in appropriately designed clinical studies.

### Immune-based therapy

Persistent hrHPV infection and lesion progression are accompanied by remodeling of the local immune microenvironment, providing a rationale for immune-based interventions. Clinical trials of E6/E7-directed therapeutic HPV vaccines have demonstrated histological regression in a subset of women with CIN2/3; however, responses remain incomplete and their optimal clinical implementation requires further validation [13, 14]. Future studies should characterize immune features associated with lesion persistence and regression and identify biomarkers capable of predicting responses to immunotherapeutic approaches. Other strategies targeting immune-checkpoint signaling or regulatory immune-cell populations remain potential avenues that require further investigation specifically in cervical precancerous lesions. The cervicovaginal microbiome may provide an additional avenue for immune modulation because dysbiosis is associated with HPV persistence, local inflammation, and altered antiviral immune responses. Future studies should determine whether microbiome-directed interventions can promote HPV clearance, restore local immune homeostasis, or reduce the risk of lesion persistence and progression.

### Multi-omics biomarker development

Biomarkers such as p16, Ki-67, DNA methylation patterns, and microRNAs have shown potential for improving diagnosis and risk stratification. However, individual biomarkers may be insufficient to capture the biological heterogeneity of cervical precancerous lesions. Integrating genomic, epigenomic, transcriptomic, and other molecular information with emerging single-cell RNA sequencing and spatial transcriptomic approaches may enable more comprehensive characterization of cellular states, intercellular interactions, and spatial organization. Such integrated signatures may ultimately improve the identification of lesions at high risk of persistence or progression. Nevertheless, prospective longitudinal validation in cervical precancerous lesions remains essential before these candidate biomarkers can be incorporated into routine clinical risk stratification.

### Interpretable AI-assisted diagnosis

Deep learning has demonstrated promising performance in cervical cytology and colposcopy, but its clinical translation requires more than improvements in diagnostic accuracy. Future studies should prioritize standardized data acquisition and annotation, multicenter external validation across diverse populations and clinical settings, and the development of explainable AI approaches that provide clinically interpretable evidence for model predictions. Improving model generalizability and transparency will be essential for building clinician confidence and integrating AI-assisted screening into routine clinical workflows.

In conclusion, advancing the management of cervical precancerous lesions will require coordinated progress in molecular targeting, immune-based therapy, multi-omics biomarker development, and interpretable AI-assisted diagnosis. Integrating these approaches with prospective clinical validation may enable more accurate risk stratification and individualized intervention, ultimately improving the prevention of cervical cancer.

## Data Availability

Not applicable.

## Abbreviations

AIS: adenocarcinoma in situ
HPV: human papillomavirus
CIN: cervical intraepithelial neoplasia
LSIL: low-grade squamous intraepithelial lesion
HIV: human immunodeficiency virus
HSIL: high-grade squamous intraepithelial lesion
CC: cervical cancer
ICC: invasive cervical cancer
CESC: cervical squamous cell carcinoma and endocervical adenocarcinoma
hrHPV: high-risk HPV
EMT: epithelial-mesenchymal transition
ECM: extra cellular matrix
p53: tumor protein p53
p16: cyclin-dependent kinase inhibitor p16
MAPK: mitogen-activated protein kinase
EGFR: epidermal growth factor receptor
RAS: rat sarcoma
RAF: rapidly accelerated fibrosarcoma
MEK: mitogen-activated protein kinase kinase
ERK1/2: extracellular signal-regulated kinase ½
PI3K: phosphoinositide 3-kinase
AKT: protein kinase B
mTOR: mammalian target of rapamycin
LRP5/6: low-density lipoprotein receptor-related protein5/6
GSK3β: glycogen synthase kinase 3β
CKIα: casein kinase Iα
TCF/LEF: T-cell factor/lymphoid enhancer factor
TNFR: tumor necrosis factor receptor
TLR: toll-like receptor
IKK: IκB kinase
TGF-β: transforming growth factorβ
CAFs: carcinoma-associated fibroblasts
Ki67: proliferation marker protein Ki-67
CDC6: cell division cycle 6
MCM5: minichromosome maintenance complex component 5
scRNA-seq: single-cell RNA sequencing
ST: spatial transcriptomics
TME: tumor microenvironment
KRT17: keratin 17
CSCC: cervical squamous cell carcinoma
CDKN2A: cyclin-dependent kinase inhibitor 2 A
TOP2A: DNA topoisomerase II alpha
mCAF: matrix-fibroblast
HCC: hepatocellular carcinoma
AVE: automated visual evaluation
ML: machine learning
NF-κB: nuclear factor kappa B
LAMs: lipid-associated macrophages
pDCs: plasmacytoid dendritic cells
CA10: carbonic anhydrase 10
DPP10: dipeptidyl peptidase like 10
HAS1: hyaluronan synthase 1
ZSCAN1: zinc finger and SCAN domain containing 1
ST6GALNAC5: ST6 N-Acetylgalactosaminide Alpha-2,6-Sialyltransferase 5
ANKRD18CP: ankyrin repeat domain 18 C, pseudogene
PAX2: paired box 2
CDH6: cadherin 6
GFRA1: GDNF family receptor alpha 1
GATA4: GATA binding protein 4
KCNIP4: potassium voltage-gated channel interacting protein 4
LHX8: LIM homeobox 8
SPP1: secreted phosphoprotein 1
NRG1: neuregulin 1
ERBB3: Erb-B2 receptor tyrosine kinase 3
NICD: notch intracellular domain
RBPJ: recombination signal binding protein for immunoglobulin
HMGB1: high-mobility group box 1
Treg: regulatory T-cell
CDX: cell-derived xenograft
